# Fibroin and Sericin from *Bombyx mori* Silk Stimulate Cell Migration through Upregulation and Phosphorylation of c-Jun

**DOI:** 10.1371/journal.pone.0042271

**Published:** 2012-07-31

**Authors:** Celia Martínez-Mora, Anna Mrowiec, Eva María García-Vizcaíno, Antonia Alcaraz, José Luis Cenis, Francisco José Nicolás

**Affiliations:** 1 Instituto Murciano de Investigación y Desarrollo Agrario y Alimentario (IMIDA), La Alberca, Murcia, Spain; 2 Laboratorio de Oncología Molecular y TGF-ß, Hospital Universitario Virgen de la Arrixaca, El Palmar, Murcia, Spain; Massachusetts General Hospital/Harvard Medical School, United States of America

## Abstract

Wound healing is a biological process directed to the restoration of tissue that has suffered an injury. An important phase of wound healing is the generation of a basal epithelium able to wholly replace the epidermis of the wound. A broad range of products derived from fibroin and sericin from *Bombyx mori* silk are used to stimulate wound healing. However, so far the molecular mechanism underlying this phenomenon has not been elucidated. The aim of this work was to determine the molecular basis underlying wound healing properties of silk proteins using a cell model. For this purpose, we assayed fibroin and sericin in a wound healing scratch assay using MDA-MB-231 and Mv1Lu cells. Both proteins stimulated cell migration. Furthermore, treatment with sericin and fibroin involved key factors of the wound healing process such as upregulation of c-Jun and c-Jun protein phosphorylation. Moreover, fibroin and sericin stimulated the phosphorylation of ERK 1/2 and JNK 1/2 kinases. All these experiments were done in the presence of specific inhibitors for some of the cell signalling pathways referred above. The obtained results revealed that MEK, JNK and PI3K pathways are involved in fibroin and sericin stimulated cells migration. Inhibition of these three kinases prevented c-Jun upregulation and phosphorylation by fibroin or sericin. Fibroin and sericin were tested in the human keratinocyte cell line, HaCaT, with similar results. Altogether, our results showed that fibroin and sericin initiate cell migration by activating the MEK, JNK and PI3K signalling pathways ending in c-Jun activation.

## Introduction

Wound healing is a complex process that includes inflammation, reepithelialization, angiogenesis and tissue remodeling with the aim to restore tissue integrity [1], [2]. Reepithelialization involves migration and proliferation of keratinocytes to cover the wounded surface [3]. One of the initial steps of cells migration is polarization and protrusion in the direction of migration [4]. The leading edge of the collective cell migration consists of intrinsically bipolar leading cells, which explore the tissue environment, find path, generate traction and, where needed, prototypically remodel extracellular matrix (ECM) for path generation. Leading cells are engaged with the tissue substrate while their rear region remains engaged with the neighbor cells [5]. In collective migration, cells retain their cell–cell adhesion and communication to collectively polarize and migrate directionally [5]. Cell migration is driven by growth factors and cytokines that are released concordantly into the injury site and it requires the integration of key events in signaling, cytoskeletal reorganization, and adhesion processes [6].

It is well known that mitogen-activated protein (MAP) kinases family members such as ERK1/2 and JNK are important for cell migration [7], [8], [9], [10], [11]. JNK signalling is important in the movement of epithelial sheets, wound healing, apoptosis, cell survival and tumour development [12]. In vitro, mammalian JNKs efficiently phosphorylate c-Jun on two serine residues (Ser63 and Ser73) in the amino-terminal domain of the protein. This phosphorylation correlates well with c-Jun activation [13]. In mammalian cells, JNK is only phosphorylated in cells at the edge of the wound and inhibition of JNK pathway blocks migration and lamelipodia extension [14].

Silk is a broad family of protein-based natural polymers secreted by diversity of arthropods. The silk produced by the lepidopteran insect *Bombyx mori* is the most recognized one due to its long use as a high value textile fiber. In recent years, additional applications have been developed for silk, mainly in the field of biotechnology and biomedicine. The versatility of these new implementations arises from the singularity of the molecular structure of silk. The fiber, secreted by the silkworm, is a continuous strand composed of two proteins of very different nature: fibroin and sericin. Fibroin constitutes the 70% of the fiber strand weight and functions as a structural component. It is composed of two equimolar protein subunits of 370 and 25 kDa covalently linked by disulfide bonds [15]. Sericin is a second type of the silk protein that glue together the fibroin threads in order to create the compact and closed structure of the cocoon [16].

There is need for new-generation biomaterials and therapies providing healing acceleration and reducing wound-related complications. A large number of patients suffer from chronic, non-healing ulcers [17]. Many studies have showed that the compounds of the silk, fibroin and sericin, have been successfully used in a therapeutic practice as a wound dressing to stimulate the healing process [18]. Fibroin and sericin seem to exert an active role in the wound healing process. The stimulating effect on wound healing has been demonstrated for sericin both in cell cultures and *in vivo* in a mice model [19], [20]. Additionally, sericin gel films have been successfully used as wound dressings [21]. On the other hand, fibroin has also shown a similar effect on wound healing [22], and in addition to that, the peptides that result from its trypsin digestion are also stimulating of fibroblast growth [23]. Biomaterials derived from fibroin have been tried with good results in wound healing after diverse elaborations such as hydrogels [24], sponges [25], films [26] and electrospun nanofiber mats [27].

Fibroin and sericin have been successfully used in therapeutic practice to accelerate tissue regeneration; however, little work at the molecular level has been reported on their biologically functional properties. In this paper we unveil some molecular aspects underlying fibroin and sericin wound healing properties. Using the human cell line MDA-MB231 as a cell model, we assayed fibroin and sericin in a wound healing scratch assay and both proteins stimulated cell migration. Fibroin and sericin stimulated the phosphorylation of ERK1/2 and JNK1/2 kinases. Additionally, treatment with fibroin and sericin upregulated *c-jun* gene expression and increased the amount of phosphorylated c-Jun. Some of these results were corroborated in Mv1Lu cells. The use of JNK specific inhibitor SP600125, PI3K specific inhibitor LY294002 or MEK specific inhibitor PD98059 prevented cell migration stimulated by fibroin or sericin. The use of these inhibitors also prevented fibroin and sericin c-Jun upregulation and phosphorylation. Finally, sericin and fibroin tested in the human keratinocyte HaCaT induce the phosphorylation of ERK1/2 and the upregulation of c-Jun. Altogether, our results showed than fibroin and sericin regulate cells migration by activating the JNK signalling pathway.

## Results

### Fibroin and Sericin Induce Cell Migration

**Figure 1:**
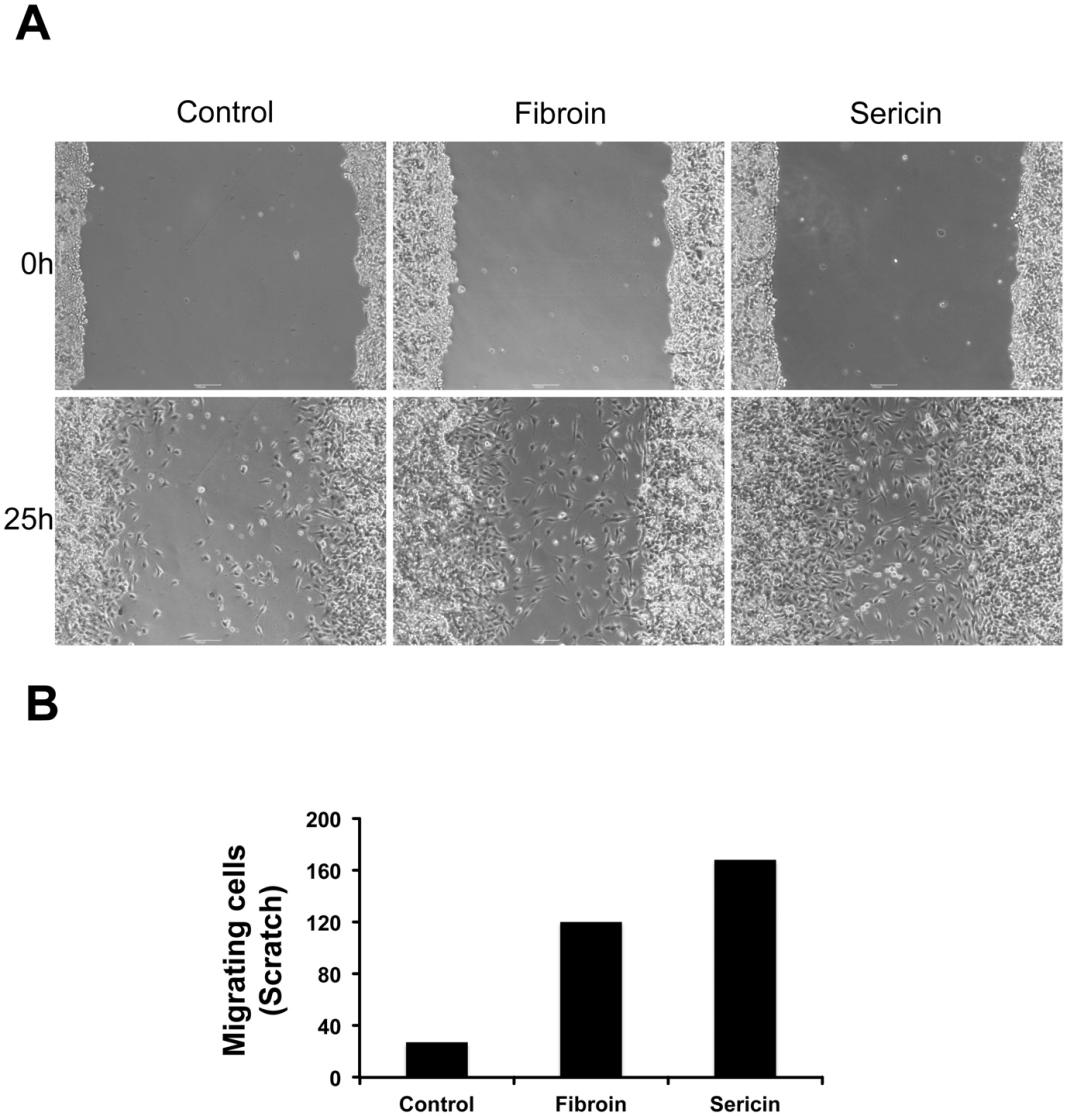
Fibroin and sericin induce motility in MDA-MB-231 cells. A. Wound healing scratch assay was made on 24 hours serum-starved, confluent MDA-MB-231 cells by drawing a line across the bottom of the dish, following by treatment with either 0.4% fibroin or 0.05% sericin. The figure shows micrographs of the extent of closure obtained under control conditions compared to those with either fibroin or sericin after 25 hours treatment. Phase-contrast microscopy pictures were taken of the wounded area. B. Cell migration quantification of pictures in A was assessed by counting the number of cells in the central gap (see Materials and Methods). Cell migration was represented as number of cells filling the central gap. The experiment was performed independently at least three times. A representative result is shown.

In this study we have investigated the molecular mechanisms that are responsible for the effect of fibroin and sericin on the wound healing process. Breast cancer MDA-MB-231 [28], [29], [30] is used in the wound healing scratch experiments to assess cell migration. We examined the effect of fibroin and sericin on migration by performing a wound healing scratch assay. Confluent culture monolayer of MDA-MB-231 was scratched and either fibroin or sericin was added to the medium. Several concentrations of fibroin and sericin were empirically tested in a migration assay on both MDA-MB-231 and Mv1Lu (see below) cell lines. The minimal amount required of either protein was 0.4% for fibroin or 0.05% for sericin to induce cell migration (data not shown). The experiment was performed in the absence of serum in order to restrain cells proliferation. The wounded area was examined by phase-contrast microscopy at the indicated times. As shown in Figure 1A and B, treatment with either fibroin or sericin significantly induced migration of MDA-MB-231 cells into the scratch area when compared to untreated cells. We used the nonmalignant mink lung epithelial cells Mv1Lu [31], [32], [33], another cell line used for the wound healing scratch test. Fibroin and sericin stimulation of Mv1Lu wound healing scratch assay showed similar results (Figure S1A and B). All together the results show that fibroin and sericin induced MDA-MB-231 or Mv1Lu cells migration.

### Fibroin and Sericin Induce ERK and JNK Signaling Pathways

**Figure 2:**
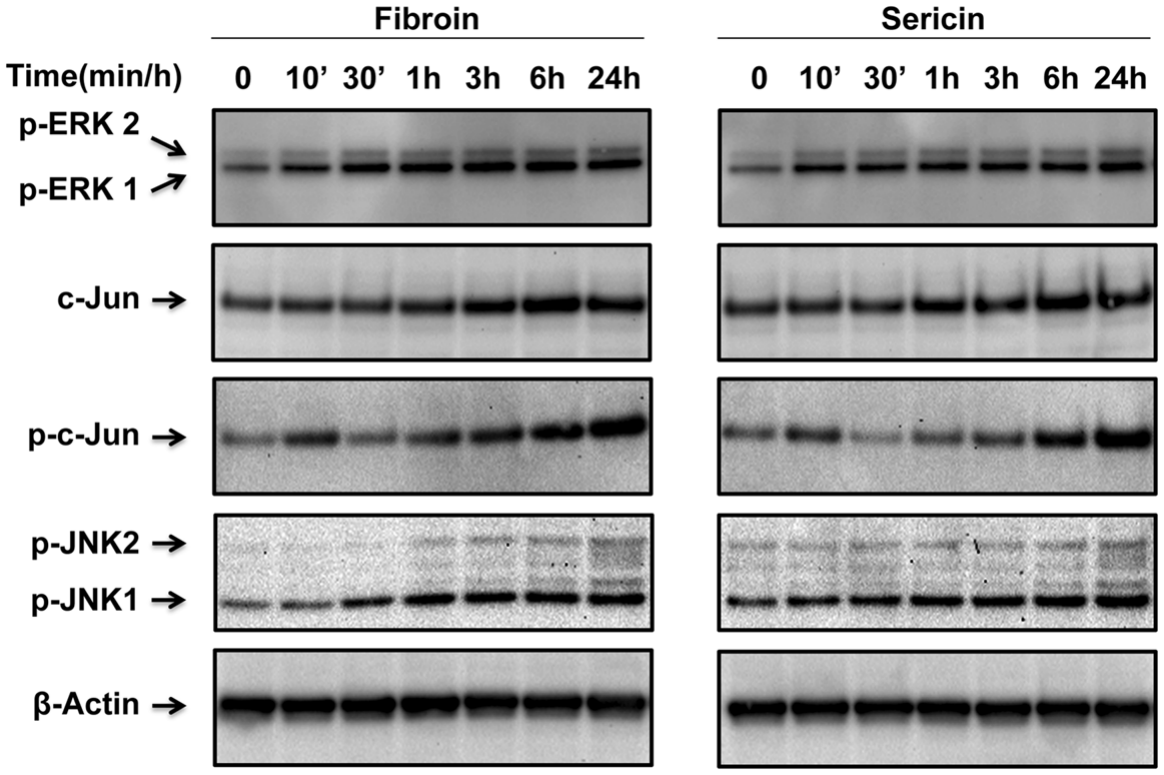
Fibroin and sericin induce phosphorylation of ERK1/2, JNK1/2 kinases and c-Jun expression and phosphorylation in MDA-MB-231 cells. A. Total cell lysates from serum-deprived, sub-confluent MDA-MB-231 cells treated with either 0.4% fibroin or 0.05% sericin for different times were analyzed by Western blot for phospho-ERK1/2, c-Jun, phospho-c-Jun, phospho-JNK1/2 proteins. ß-Actin was used as a protein loading control.

It is well known that mitogen-activated protein (MAP) kinases family members such as ERK and JNK are important for regulation of cell migration. To investigate whether ERK and JNK signalling pathways were involved in the stimulatory effect of fibroin and sericin on cell migration, we studied changes in different proteins expression and phosphorylation of total protein from sub-confluent MDA-MB-231 cells stimulated for various periods of time with either fibroin or sericin. Fibroin and sericin were able to increase the c-Jun protein expression and its phosphorylation in a time dependent manner. Strikingly, the phosphorylation of c-Jun was acutely increased after 10 minutes of both fibroin and sericin treatment. With further time, c-Jun phosphorylation increased steadily (Figure 2). Consistently, treatment of MDA-MB-231 cells with either fibroin or sericin induced a time dependent increase in phosphorylation of JNK1/2 kinases (Figure 2). All JNK kinases phosphorylation, c-Jun phosphorylation and c-Jun protein expression were more apparent 24 hours after fibroin or sericin treatment. Finally, treatment with fibroin or sericin also induced the phosphorylation of ERK1/2 kinases (Figure 2). Thus, the results showed that fibroin and sericin activate ERK and JNK signalling pathways and induce the upregulation and phosphorylation of c-Jun.

### Fibroin and Sericin Induce Expression of a Gene Involved in Cell Migration

**Figure 3:**
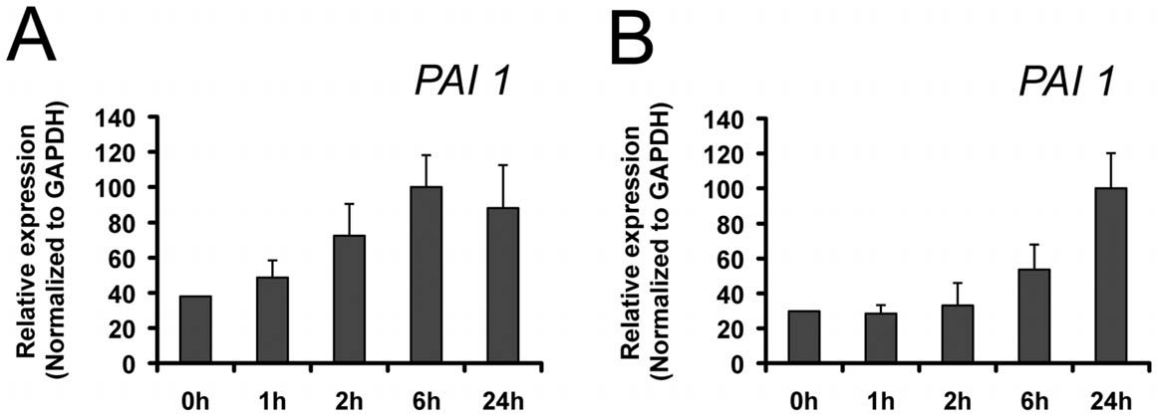
Fibroin and sericin induce the expression of PAI-1, a gene involved in motility. A. RNAs from MDA-MB-231 cells treated with fibroin for indicated times were analyzed by qPCR for *PAI-1* gene. B. RNAs from MDA-MB-231 cells treated with sericin for indicated times were analyzed by qPCR for *PAI1* gene. Gene expression is represented as a ratio to *GAPDH*.

It has been shown that JNK leads to the induction of downstream target genes, e.g. Plasminogen Activator Inhibitor 1 (*PAI-1*) [34]. PAI-1 was shown to be important for migration, in addition to its role in proteolysis control [35]. In order to better understand the stimulatory effect of fibroin and sericin on cell migration, we studied their effect on the expression of *PAI1*. Real-time PCR analyses showed that MDA-MB-231 cells exhibited a time-dependent increase of *PAI-1* expression over 24 hours after fibroin (Figure 3A) or sericin treatment (Figure 3B). The expression of *PAI-1* has a rapid response upon fibroin or sericin stimulation in MDA_MB-231 cells, about 1 to 2 hours, and its expression was sustained over a 24 hours period (Figure 3A, B). Our data showed that the stimulation of MDA-MB-231 cells with either fibroin or sericin induces the expression of a gene involved in cell migration.

### Fibroin and Sericin did not Affect Proliferation of MDA-MB-231 and Mv1Lu Cells

**Figure 4:**
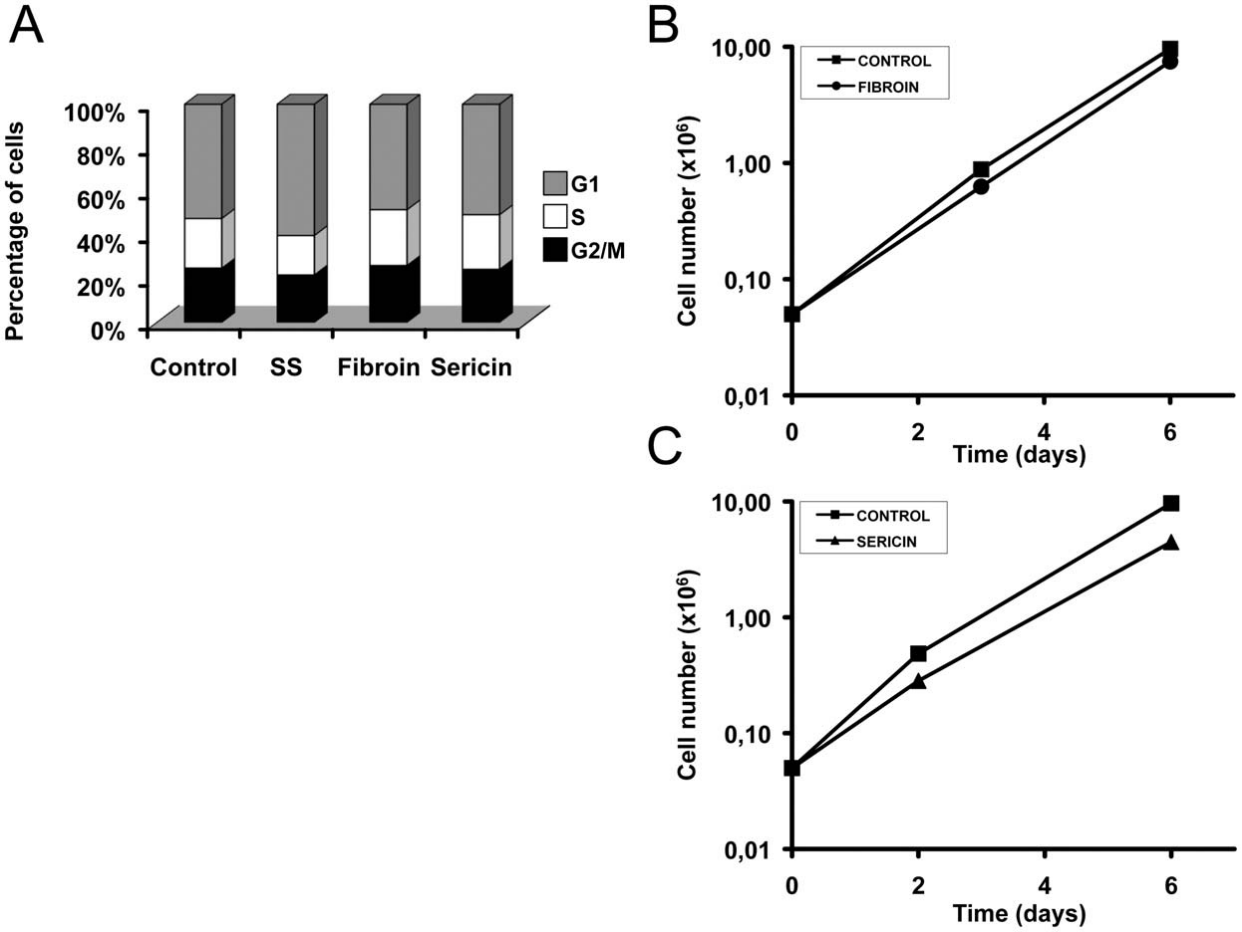
Fibroin and sericin do not affect cell cycle distribution or proliferation of MDA-MB-231. A. MDA-MB-231 cells were treated with either 0.05% sericin or 0.4% fibroin, or serum starved (indicated as SS) for 24 hours. Cell cycle distribution was measured using fluorescence activated cell sorting (FACS). Data are presented as percentage of cells at the different phases of cell cycle. B. Effect of fibroin on MDA-MB-231 cells proliferation was assessed by counting cells at the indicated days. The logarithm of the mean number of cells is plotted against time. C. Effect of sericin on MDA-MB-231 cells proliferation was assessed by counting cells at the indicated days. The logarithm of the mean number of cells is plotted against time.

As the activation of JNK1/2 and ERK1/2 may have an effect on proliferation and cell survival [36], we investigated the effects of sericin and fibroin on cell cycle in MDA-MB-231 cells. Analysis of cell cycle was performed by flow cytometry after treatment of cells with medium supplemented with either fibroin or sericin. After 24 hours treatment, the cell fraction of all tested cells in G2 and S phase was not increased significantly, showing that fibroin and sericin did not have substantial effect on the cell cycle distribution in MDA-MB-231 (Figure 4A). Similarly, fibroin or sericin did not have any effect on cell cycle when assayed on Mv1Lu cells (Figure S2A). The same results were obtained after 48 hours treatment with fibroin or sericin (data not shown). As proliferation of eukaryotic cells is a highly regulated process that may not be reflected in the different distribution of phases of the cell cycle, we determined the time-dependent effect on proliferation of fibroin or sericin on MDA-MB-231 cells. The results showed that fibroin and sericin did not have substantial effect on MDA-MB-231 cell proliferation (Figure 4B, C). Similar results were found when fibroin and sericin were assayed on Mv1Lu cells (Figure S2B, C). Thus, the results showed that fibroin and sericin have only little effect on the proliferation of MDA-MB-231 and Mv1Lu cells.

### MEK, JNK and PI3K Inhibitors Prevent Fibroin- and Sericin-induced Cell Migration

**Figure 5:**
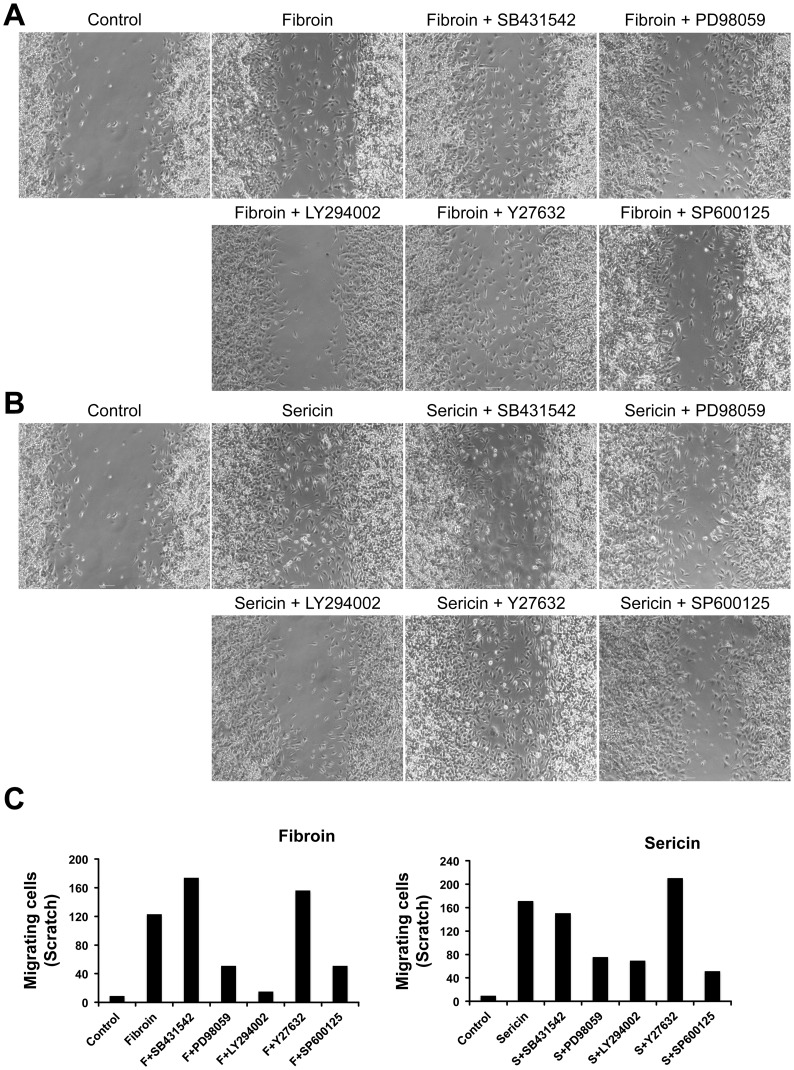
MEK, PI3K and JNK inhibitors prevent fibroin and sericin cells migration stimulation. Wound healing scratch assay was made on 24 hours serum-starved confluent MDA-MB-231 cells that were treated with either 0.4% fibroin or 0.05% sericin for 25 hours, in the absence or presence of following inhibitors: SB431542, PD98059, LY294002, Y27632 and SP600125. A. Micrographs of the extent of closure obtained after 25 hours in the control sample or samples treated with fibroin supplemented or not with the indicated inhibitors. B. Micrographs of the extent of closure obtained after 25 hours in the control sample or samples treated with sericin supplemented or not with the indicated inhibitors. In all cases, the wounded area was examined by phase-contrast microscopy. C. Cell migration quantification of different samples in A and B was assessed by counting the number of cells in the central gap (see Materials and Methods). Cell migration was represented as number of cells filling the central gap. The experiment was performed independently at least three times. A representative result is shown.

We have shown that fibroin and sericin upregulated *c-jun* transcription and stimulated c-Jun phosphorylation. In order to investigate it further, we performed a wound healing scratch assay on MDA-MB-231 cells with fibroin or sericin. Well-known inhibitors of various signaling pathways related to cell migration were used: PD98059 (MEK inhibitor), LY294002 (PI3K inhibitor), Y27632 (ROCK inhibitor) and SP600125 (JNK inhibitor). After 24 hours, the wounded area was examined by phase-contrast microscopy. Fibroin or sericin stimulation of Mv1Lu cell migration was prevented by PD98059, LY294002 and SP600125, but not by Y27632 (Figure S3A, B, C). We performed the same experiment on MDA-MB-231 cells. Similarly, PD98059, LY294002 and SP600125 inhibited fibroin and sericin induced MDA-MB-231 cell migration into the gap. Additionally, fibroin and sericin induced MDA-MB-231 cells migration was not inhibited by Y27632 treatment (Figure 5A, B, C). In response to fibroin and sericin we detected an stimulation of the expression of *PAI1,* a TGFβ-dependent gene (*PAI1*) [37]. As TGFß stimulates cell migration [38], we investigated the possible involvement of TGFβ signalling in fibroin- and sericin-induced cell migration by using specific inhibitor SB431542. Both fibroin and sericin cell migration stimulation was maintained in the presence of SB431542 suggesting that TGFß signaling is not involved in fibroin or sericin induced cell migration in both Mv1Lu and MDA-MB-231 cells (Figure S3A, B C; Figure 5A, B, C). Altogether, these s strongly suggest that fibroin and sericin induce cell migration with the involvement of active MEK, JNK and PI3 kinases.

### Fibroin and Sericin Upregulated c-Jun Transcription and Induced c-Jun Phosphorylation through MEK, JNK and PI3K Signaling Events

**Figure 6:**
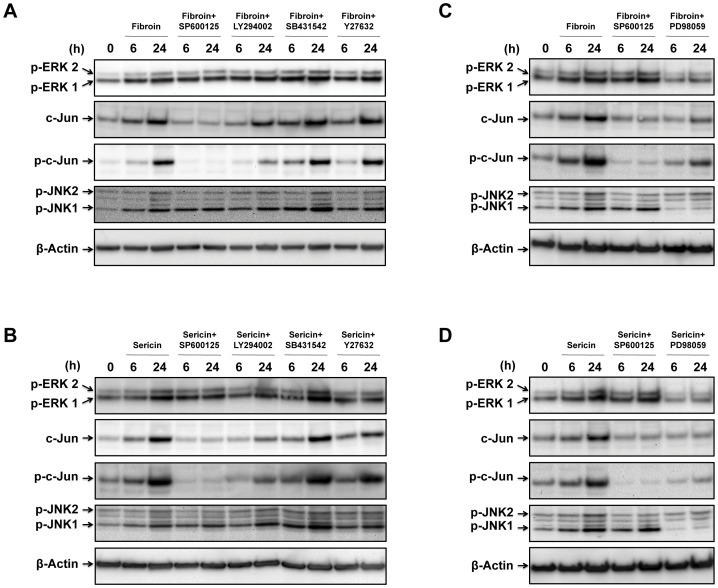
Fibroin and sericin induced c-Jun transcription and phosphorylation are prevented by JNK, PI3K or MEK inhibitors. For A and B, serum-deprived, sub-confluent MDA-MB-231 cells were treated for different times with either 0.4% fibroin or 0.05% sericin in the absence or presence of the following inhibitors: SP600125, LY294002, SB431542 and Y-27632. A. Fibroin treated total cell lysates were analyzed by Western blot for phospho-ERK1/2, c-Jun, phospho-c-Jun, phospho-JNK1/2 proteins. ß-Actin was used as a protein loading control. B. Sericin treated total cell lysates were analyzed by Western blot for phospho-ERK1/2, c-Jun, phospho-c-Jun, phospho-JNK1/2 proteins. ß-Actin was used as a protein loading control. For C and D, serum-deprived, sub-confluent MDA-MB-231 cells were treated for different times with either 0.4% fibroin or 0.05% sericin in the absence or presence of the following inhibitors: SP600125 and PD98059. C. Fibroin treated total cell lysates were analyzed by Western blot for phospho-ERK1/2, c-Jun, phospho-c-Jun, phospho-JNK1/2 proteins. ß-Actin was used as a protein loading control. D. Sericin treated total cell lysates were analyzed by Western blot for phospho-ERK1/2, c-Jun, phospho-c-Jun, phospho-JNK1/2 proteins. ß-Actin was used as a protein loading control.

The observation that MEK, JNK and PI3K inhibitors were able to prevent fibroin- and sericin-induced migration (see Figure 5, Figure S3) prompted us to investigate the effect of those inhibitors on fibroin and sericin induced upregulation and phosphorylation of c-Jun. We performed Western blot of total protein lysates from sub-confluent MDA-MB-231 cells treated with either fibroin or sericin in the presence of several inhibitors: SP600125 (JNK inhibitor), LY294002 (PI3K inhibitor), SB431542 (ALK5 inhibitor) and Y27632 (ROCK inhibitor). Fibroin and sericin induced the stimulation of ERK1/2 phosphorylation that was unaffected by the presence of any of the above inhibitors. Moreover, stimulation of JNK1/2 phosphorylation by either fibroin or sericin was not impeded by any of the inhibitors (Figure 6A and 6B). However, the upregulation of c-Jun expression by fibroin was totally prevented by SP600125 and partially affected by LY294002, whereas SB431542 or Y27632 had no effect on it (Figure 6A). Similarly, fibroin-induced stimulation of c-Jun phosphorylation was completely impeded by SP600125 and partially inhibited by LY294002, although this may reflect the low levels of c-Jun expression in this samples. Moreover, SB431542 and Y27632 inhibitors had no effect (Figure 6A). Very similar results were found for sericin c-Jun expression upregulation and c-Jun phosphorylation stimulation (Figure 6B). As fibroin or sericin had a stimulatory effect on the phosphorylation of ERK1 and 2, we tested MEK inhibitor PD98059. The presence of PD98059 prevented ERK1/2 phosphorylation in response to fibroin or sericin treatment (Figure 6C, D); moreover, JNK1 phosphorylation was severely affected by the presence of PD98059, although JNK2 phosphorylation was not. Clearly sericin c-Jun transcription upregulation was prevented by PD98059, and the lack of phosphorylation of c-Jun could be a consequence of their low expression (Figure 6D). However, treatment of PD98059 on fibroin-induced c-Jun phosphorylation or upregulation was less inhibitory (Figure 6 C). Altogether, these results show that fibroin and sericin induced transcription upregulation and phosphorylation of c-Jun. Additionally, data with MEK1, JNK and PI3K inhibitors indicate the participation of these signaling elements in fibroin and sericin cell migration response through the activation of c-Jun.

### c-Jun is Directly Involved in Fibroin- and Sericin-induced MDA-MB-231 Cells Migration

**Figure 7:**
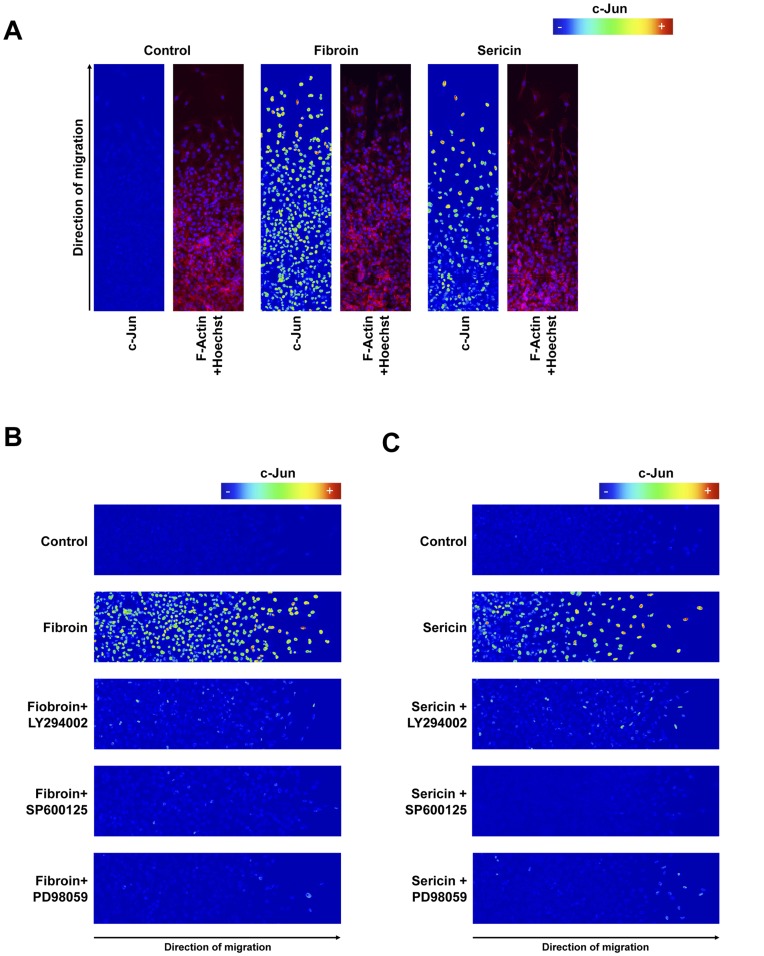
c-Jun is directly involved in fibroin- and sericin-induced migration of the MDA-MB-231 cells. Monolayer of confluent MDA-MB-231 cells, serum-deprived for 24 hours, was subjected to a wound healing scratch assay with fibroin and sericin treatments and inhibitors in the indicated combinations. After 25 hours the cells were fixed, permeabilized and immunostained against c-Jun (pseudo-color) and co-stained with phalloidin for F-Actin (red). Nuclei were revealed with Hoechst 33258 (blue). Representative images containing wound’s healing border (the leading edge) were taken with confocal microscope LSM 510 META from ZEISS. All images were acquired by confocal microscopy using the same settings for all the conditions, so c-Jun protein levels could be compared among different treatments. For clear visualization of c-Jun protein expression level, a pseudo-color code ranging from red (highest level expression) to blue (lowest expression level) was used. The arrows at the bottom indicate the direction of migration of the cells. A. Immunostaining of c-Jun at the leading edge of the MDA-MB-231 cells treated with either 0.4% fibroin or 0.05% of sericin. B. Immunostaining of c-Jun at the leading edge of the MDA-MB-231 cells treated with 0.4% fibroin or 0.4% fibroin with the following inhibitors: LY294002 (PI3K inhibitor) SP600125 (JNK inhibitor) and PD98059 (MEK inhibitor). C. Immunostaining of c-Jun at the leading edge of the MDA-MB-231 cells treated with 0.05% sericin or 0.05% sericin with the following inhibitors: LY294002 (PI3K inhibitor) SP600125 (JNK inhibitor) and PD98059 (MEK inhibitor). The experiment was performed independently at least three times. A representative result is shown.

Treatment of MDA-MB-231 cells with either fibroin or sericin stimulates cells migration. We observed that fibroin and sericin upregulate the expression of c-Jun and stimulate phosphorylation of c-Jun (see Figure 2) that was prevented by JNK, PI3K and MEK inhibitors (see Figure 6). Additionally JNK, PI3K and MEK inhibitors were able to block fibroin- and sericin-induced cell migration (see Figure 5). This data strongly suggest the involvement of c-Jun as a key molecule responsible for the stimulatory effect of fibroin and sericin in cell migration. In mammalian cells, JNK is phosphorylated only in cells at the edge of the wound [39]. Additionally c-Jun, a component of the AP-1 transcription factor complex, is essential for the organization of the epidermal leading edge in wound healing [40]. We decided to test whether the effect of fibroin and sericin during re-epithelisation was associated with the expression of c-Jun at the wound border. Thus, we assayed c-Jun protein expression by immunofluorescence at the wound-healing border of the wound healing scratch assay of MDA-MB-231 cells stimulated with either fibroin or sericin. To better quantify c-Jun expression, fluorescence derived from c-Jun was pictured as a rainbow quantifying scale. Upon fibroin or sericin stimulation, we observed a strong upregulation of c-Jun (Figure 7A). Such upregulation was even more pronounced as the cells are situated closer to the wound border of the wound scratch (Figure S4A, B, C). Interestingly, the addition of LY294002, SP600125 and PD98059 prevented the fibroin stimulation of c-Jun (Figure 7B). Similarly, LY294002, SP600125, and PD98059 blocked sericin induced c-Jun expression (Figure 7C). These data suggest that cell migration stimulation of fibroin and sericin is directly related to the expression of c-Jun at the wound healing edge.

### Fibroin and Sericin Induce MEK and JNK Signaling Pathway in Human Spontaneously Immortalized Keratinocytes

**Figure 8:**
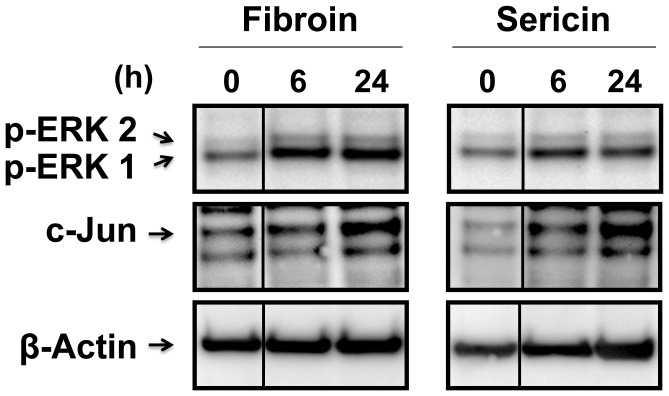
Fibroin and sericin induce phosphorylation of ERK1/2 and upregulation of c-Jun in keratinocytes. Immunoblot of equal amounts of total cell lysates from serum-deprived HaCaT cells treated with either 0.4% fibroin or 0.05% sericin for the indicated times for the indicated proteins. ß-Actin was used as a protein loading control. Line between times 0 and 6 of the western blots indicates that two distant parts of the same gel were pasted together.

As keratinocytes are directly involved in the re-epithelialization phase of the wound healing process [41], we studied the effect of fibroin and sericin on the human keratinocyte cell line HaCaT which is widely used as a model for keratinocyte responses [42]. Stimulation with fibroin and sericin induced phosphorylation of ERK1/2 (Figure 8). Moreover, both fibroin and sericin were able to upregulate c-Jun protein (Figure 8). The expression of c-Jun was most pronounced 24 hours treatment. These data suggest that fibroin and sericin stimulation have similar consequences in human keratinocytes for c-Jun expression.

## Discussion

In the present study we have investigated the molecular mechanisms that may be responsible for the effect of fibroin and sericin on wound healing process. Our results have shown that fibroin and sericin, added as a supplement to the cell culture, induced cell migration. We have found that fibroin and sericin were able to increase phosphorylation of ERK1/2, c-Jun and JNK1/2, as well as increase of c-Jun expression. Similar results were obtained on HaCaT cells. Neither cells proliferation nor cell cycle was affected by fibroin or sericin treatments. The presence of fibroin or sericin stimulated the expression of *PAI1* a gene related to cell migration. MEK, JNK and PI3K inhibitors prevented fibroin and sericin stimulation of cell migration. Moreover, inhibition of MEK, JNK and PI3K pathways impeded fibroin- and sericin-induced transcription upregulation of c-Jun and as a consequence we found less phosphorylated-c-Jun. Additionally, MEK, JNK and PI3K inhibitors prevented fibroin- and sericin-induced c-Jun expression at the leading edge of MDA-MB-231 migrating cells.

Fibroin has been reported as a negative regulator of cell proliferation in ECV304 and mouse fibroblast cells line L929 [43]. In contrast, fibroin prepared from fresh cocoon fibers enhanced human skin fibroblasts proliferation [44]. On the other hand, sericin enhanced the attachment and growth of mouse fibroblasts, when used as substratum [18]. Similar effect on growth promotion of several human cell lines and mouse hybridoma was found when sericin was added to culture medium [45]. Other author attributed sericin-dependent improvement in proliferation of primary human skin fibroblasts to the enhanced initial attachment of the cells [46]. In wound healing, different cell types are designed to migrate and proliferate. Keratinocytes at the leading edge migrate over the injured dermis, whereas only the keratinocytes that are behind the leading edge proliferate and mature [41], [47]. Our results showed that treatment of MDA-MB-231 and Mv1Lu cells with fibroin and sericin induced cell migration in wound healing scratch assay. Additionally, fibroin and sericin did not affect either cell proliferation or cell cycle distribution.

There is evidence that different mitogen-activated protein kinase (MAPKs) family members are involved in the regulation of cell migration [7], [8]. The MAPKs family consists of extracellular signal-regulated kinases 1 and 2 (ERK1/2), p38 and c-Jun N-terminal kinases 1 and 2 (JNK1/2) [9]. JNK modulate motility by regulation of transcription, e.g. via activation of c-Jun, an AP-1 transcription factor [17], [48], [49]. Our results showed that fibroin and sericin were able to induce phosphorylation of both JNK1/2 and c-Jun as well as an increase of c-Jun protein expression in sub-confluent MDA-MB-231 cells. JNK-c-jun signaling pathway leads to the induction of several downstream target genes; one of which is the type 1 plasminogen activator inhibitor (*PAI-1*) [34], [50]. During wound healing, elevated levels of PAI-1 protect ECM proteins from proteolytic degradation, which helps to accelerate wound healing [51], [52]. Additionally, PAI-1 plays a dual role in wound healing, firstly it stimulates migration, facilitating the re-epithelialization of the wound bed, and secondly it promotes successful cellular attachment [35], [53], [54]. Keratinocytes from PAI-1 knockout mice had a significant wound-healing defect [55]. We have shown that the presence of fibroin or sericin in MDA-MB-231 cells culture medium stimulated the expression of *PAI1*. Thus, the stimulation of *PAI1* by fibroin and sericin may promote successful cell attachment during cell migration and facilitate the re-epithelialization of wounds. Although it is well established that *PAI1* expression is upregulated by TGFβ [56], our data indicate that TGFβ signaling pathway is not involved in fibroin- or sericin-induced migration. Altogether, fibroin and sericin induce MDA-MB-231 cells migration through the upregulation of critical genes such as *c-JUN*.

Activated JNK plays a critical role in the migration of fibroblasts in wound healing assays [48]. c-Jun was found to be important in conditions where the proliferation and motility of epithelium are rapidly altered, such as eyelid fusion and wound healing [10]. Wounds in c*-jun* conditional knockout mice showed a severe delay in healing as a result from a reduced migratory capability of keratinocytes [10]. Inhibitor of JNK, SP600125, has been shown to restrain the migration of numerous cell types [10], [48]. We have shown that SP600125 was very effective in inhibiting either fibroin- or sericin-induced scratch wound closure, showing a strong link between cell migration mediated by fibroin and sericin and JNK pathway. SP600125 was also able to prevent fibroin- and sericin-induced protein expression and phosphorylation of c-Jun. It has been shown that upregulation of c-Jun in cells at the wound leading edge is associated with the process of wound healing [40], [57]. Additionally, keratinocytes lacking c-Jun are unable to migrate or elongate properly in culture at the border of wound healing scratch assays [40]. Our data showed that fibroin and sericin stimulated migration and induced c-Jun expression at the collective cell migration leading edge. Consistently, JNK inhibitor prevention of MDA-MB-231 cells migration correlated with the absence of c-Jun expression at the wound border leading edge. Thus, c-Jun appears to be one of the key molecules in the cell migratory response to fibroin and sericin.

ERK1/2 activation regulates migration during wound healing [58]. Indeed in our hands, treatment of MDA-MB-231 or Mv1Lu with EGF induced migration of these cells. EGF was found to have stimulatory effect on corneal epithelial wound repair by increasing migration [59]. Moreover, EGF effect on cell migration has been related to the phosphorylation of JNK by MEKK1 [60], [61]. MEKK1 preferentially activated JNK1 and influences the activity of ERK [61]. Activation of JNK is a critical step to mediate fundamental stages of cells migration [12]. Strikingly, our data indicate that MEK1 is upstream of both JNK1 and ERK1/2 phosphorylation upon fibroin and sericin induction in MDA-MB-231 cells. Our results showed that PD98059, an inhibitor for MEK1, prevented fibroin- or sericin-induced cell migration and this correlated well to the inhibition of ERK1/2 and JNK1 phosphorylation. PD98059 also inhibited c-Jun upregulation in response to sericin, however had a partial inhibitory effect on fibroin-induced upregulation of c-Jun. As a consequence of c-Jun upregulation changes, final phosphorylated amount of c-Jun was affected by PD98059. Fibroin- and sericin-induction of ERK1/2 phosphorylation is independent of JNK activation because treatment with SP600125 did not affect ERK1/2 phosphorylation. Altogether, these data strongly suggest an involvement of MEK1 in the fibroin- and sericin-induced activation of JNK1 required for MDA-MB-231 cell migration. Additionally, they suggest a different MEK1 requirement for the fibroin- and sericin-induced c-Jun activation.

Class I phosphoinositide 3-kinases (PI3Ks), have been considered key players in gradient sensing, leading edge definition, cell polarization, and migration [62], [63]. It has been previously published that inhibition of PI3K impairs cell migration of several cells lines [64], [65], [66]. LY294002 inhibited fibroin- and sericin-induced cell migration, suggesting the mediating role of PI3K in this response. Moreover, LY294002 inhibited c-Jun protein expression at the leading edge of the collective cell migration and partially inhibited expression and subsequently the phosphorylation of c-Jun protein in sub-confluent cell culture, pointing out a role of PI3K pathway in fibroin and sericin induced c-Jun expression during migration. The crosstalk between PI3K and JNK pathway has been shown in airway epithelial cells where PI3K activates Rac1 and finally leads to JNK activation and cell migration [66]. Both JNK and PI3K are key mediators of fibroin and sericin induced cell migration in MDA-MB-231 and Mv1Lu cells. PI3K, however, may not be mediating all JNK activation, since PI3K inhibition was not sufficient to prevent all fibroin or sericin-induced c-Jun upregulation and phosphorylation. Results with both LY294002 and PD98059 inhibitors suggest different recruitments of PI3K and MEK1 signaling pathways for the final required c-Jun activation in cell migration. This also suggests that the molecular mechanism underlying the cell response to fibroin and sericin are not the same and may differ in some of its constituents. More research need to be conducted to identify the fibroin and sericin protein domains and cell receptors that are used to trigger the complex signaling events that promote mammalian cells migration.

In conclusion, our data reveal that in mammalian cells c-Jun is a downstream mediator of fibroin- and sericin-induced migration. Furthermore, MEK1, PI3K and JNK are signaling key events of fibroin- and sericin-induced cell migration and their actions occur through downstream mechanisms leading to c-Jun activation.

## Materials and Methods

### Preparation of Fibroin and Sericin

Silkworms (*Bombyx mori*), from an autochthonous race from Murcia, Spain, producing white cocoons were reared. After the cocoon was formed, pupae were extracted and empty cocoons were boiled twice for 45 min in an aqueous solution of 0.02 M Na_2_CO_3_ (Acros Organics). This solution, containing the sericin fraction of the silk, was dialyzed for three days in deionized water in a 3.500 MW cutoff membrane. The remaining fibroin, separated from sericin solution by filtration, was rinsed thoroughly with water to clean it completely from the sericin proteins. The fibroin mat was then dried at room temperature for 72 h and dissolved in 9.3 M LiBr (Acros Organics) for 3h at 60°C to generate a 20% w/v solution. Then, it was dialyzed in distilled water for 3 days and the resultant aqueous solution was freeze dried and stored at 4°C, in order to have the purified silk fibroin ready to be dissolved in the desired concentration of deionized water. The purity of fibroin and sericin was determined by mass spectroscopy that revealed 99% of fibroin and sericin in prepared samples.

### Cell Culture and Wound Healing Scratch Assay

Human Mammary Gland cells (MDA-MB-231) [28], [29], [30] and the Human spontaneously immortalized Keratinocyte cell line (HaCaT) [42] were grown in Dulbecco’s Modified Eagle Medium (DMEM); Mink Lung Epithelial (Mv1Lu) [31], [32], [33] cells were grown in Eagle’s Minimum Essential Medium (EMEM). Both media were supplemented with 10% Fetal Bovine Serum (FBS), 1% Penicillin/Streptomycin and 1% L-Glutamine (Lonza). Cells were incubated in a humidified atmosphere at 37°C with 7.5% CO_2_.

For wound healing scratch assay, 95% confluent cells were detached by 0.05% trypsin/EDTA (Lonza) treatment and seeded for cell expansion on either 50 mm diameter plates or glass coverslips. Cells were grown for five days, then growth medium was changed for complete DMEM deprived of FBS (0% FBS) for MDA-MB-231 cells or complete EMEM deprived of FBS (0% FBS) for Mv1Lu cells for 24 hours before wound healing scratch assay. Wound was made by scratching a line across the bottom of the dish on a confluent cells monolayer using a sterile p-200 pipette tip. Cells were rinsed very gently with PBS and then the cells were cultivated in the corresponding medium deprived of serum supplemented with either 0.4% fibroin or 0.05% sericin. The following pharmacological inhibitors: LY294002 (PI3K inhibitor), 50 µM; SP600125 (JNK inhibitor), 15 µM; SB431542 (ALK5 inhibitor), 10 µM; Y27632 (ROCK inhibitor), 1 µM; or PD98059 (MEK inhibitor), 50 µM (all from Sigma); were used as supplements to the medium where indicated. Pictures were taken at 10x magnifications using an MOTIC (AE31) microscope equipped with a digital camera. To quantify migration of the wound healing scratch assay two different approaches were followed depending on the cell line used. For MDA-MB-231 cells, cells invading a central strip were counted in either the control or the different treated samples. Then, the cell number was represented for each sample. For Mv1Lu cells, the area of the gap obtained by scratching a line across the bottom of the dish was quantified using ImageJ software (http://rsbweb.nih.gov/ij/). After each treatment, the area of the same gap was measured again. The difference between initial and final areas was calculated. We interpreted bigger differences as a reflection of better migration. That difference was represented in each treatment.

### Western Blots

Sub-confluent cells (50%) serum-deprived for 24 hours were treated with either 0.4% of fibroin or 0.05% of sericin and in the presence or absence of the following pharmacological inhibitors: LY294002, SP600125, SB431542, Y27632 or PD98059 for indicated times. Cells were harvested and lysed with 20 mM TRIS pH 7.5, 150 mM NaCl, 1 mM EDTA, 1.2 mM MgCl2, 0.5% Nonidet p40, 1 mM DTT, 25 mM NaF and 25 mM β-glycerophosphate supplemented with phosphatase inhibitors (I and II) and protease inhibitors (all from Sigma). The extracts were analyzed by SDS-PAGE followed by Western blot using the appropriate antibodies. Proteins were revealed by the ECL kit (GE, Healthcare), acquired by ChemiDoc XRS (Bio-Rad).

### qPCR

For gene expression assay, RNA from 24 hours serum-deprived sub-confluent cells treated with either 0.4% fibroin or 0.05% sericin was extracted using RNeasy-mini kit (Qiagen). Typically, 1 µg of RNA was retrotranscribed with the iScript cDNA synthesis kit (Bio-Rad), and resulting cDNA was used for qPCR following SYBR premix ex Taq kit (Takara) according to manufacturer instructions. Primers used for gene amplification were: CATGGGGCCATGGAACAAGG (PAI-1-F); CTTCCTGAGGTCGACTTCAG (PAI-1-R); ACCACAGTCCATGCCATCAC (GAPDH-F); TCCACCACCCTGTTGCTGTA (GAPDH-R); [37]. PAI-1 gene expression was referred to as a ratio to GAPDH expression. Gene expression values were represented as a percentage of the sample with the highest value.

### Cell Cycle Analysis and Proliferation Assay

For cell cycle analysis, exponentially growing cells in culture were incubated for 24 hours with different concentrations of either fibroin or sericin. Then, cells were detached by trypsinization, centrifuged and the pellet of cells was immediately fixed with ice cold 70% ethanol. Cells were washed three times with cold PBS to remove ethanol and were finally stained for cell cycle with propidium iodide using a standard method. Cells were analyzed by flow cytometry using a FACSCalibur 1 (Becton Dickinson). Cell proliferation was evaluated by cell counting. Briefly, cells were seeded in a 50-mm dish at a density of 5×10^4^ cells/plate in complete medium and treated with either 0.4% fibroin or 0.05% sericin in duplicate. At the indicated days, control and treated cells were trypsinized and harvested. Cell count was performed using a TM10™ automated cell counter (Bio-Rad) and viability of cells was determined by trypan blue dye exclusion assay. Data were plotted as a growth curve.

### Immunostaining of Wound Healing Scratch Assay

Cells were grown on coverslips until they reached 100% confluence, then the cells were deprived of serum for 24 hours and wound healing scratch assay was performed with the appropriate treatment. After 25 hours, cells were fixed for 10 min with 4% formaldehyde/PBS at room temperature and were permeabilized with 0.3% Triton X-100 in PBS for 15 min. Then, fixed cells were incubated with blocking buffer (0.3% bovine serum albumin, 10% FBS, 0.1% Triton X-100 in PBS) supplemented with 5% skim milk for half an hour at room temperature. Subsequently, samples were incubated for 2 hour with primary c-Jun antibody and phalloidin diluted in blocking buffer, and then washed three times with 0.1% Triton X-100/PBS for 10 minutes. Then, cell samples were stained with the fluorescent-labeled secondary antibody together with Hoestch 33258 (Fluka, Biochemika) that was used for nuclei detection. Samples were examined and representative images were taken with confocal microscope LSM 510 META from ZEISS using the same settings in order to make different samples of the same experiment comparable among them. For quantification of the expression of c-Jun, the migration front was divided in five adjacent sectors. The total fluorescence of each sector was quantified using ImageJ software (http://rsbweb.nih.gov/ij/): initially, total amount of fluorescence of the sector was calculated by multiplying the obtained average fluorescence by the area of the sector. Then, total amount of fluorescence of the sector was divided by the number of nuclei in that sector to obtain the averaged fluorescence per nuclei. The data obtained from all the sectors was made relative to the biggest value of any of the experiment compared that was given the arbitrary value 100. This was done to be able to compare control and treatment data between them. Finally, quantification for each sector was represented as arbitrary units.

### Antibodies

The following commercial antibodies were used: anti-c-Jun, anti-βActin (both from Santa Cruz Biotechnology); anti-phospho-ERK1/2, anti-phospho JNK1 and 2, anti-phospho-c-Jun (all from Cell Signaling Technology). Alexa Fluor 594-labelled phalloidin (Molecular Probes, Invitrogen) was used to reveal Actin filaments.

## Supporting Information

Figure S1 **Fibroin and sericin induce motility in Mv1Lu cells.** Wound healing scratch assay was made on 24 hours serum-starved, confluent Mv1Lu cells by drawing a line across the bottom of the dish, following by treatment with either 0.4% fibroin (A) or 0.05% sericin (B). The figure shows micrographs of the extent of closure obtained under control conditions compared to those with either fibroin or sericin after 24 hours treatment respectively. Phase-contrast microscopy pictures were taken of the wounded area. Migration quantification was done by measuring the gap area at 0 h and after treatment. Cell migration was calculated and represented as the difference between the scratch area before treatment and the scratch area after treatment (see Materials and Methods). The experiment was repeated at least three times. A representative result is shown.
(TIF)

Figure S2 **Fibroin and sericin do not affect cell cycle distribution or proliferation of Mv1Lu cells.** A. Mv1Lu cells were treated with either 0.05% sericin or 0.4% fibroin, or serum starved (indicated as SS) for 24 hours. Cell cycle distribution was measured using fluorescence activated cell sorting (FACS). Data are presented as percentage of cells at the different phases of cell cycle. B. Effect of fibroin on Mv1Lu cells proliferation was assessed by counting cells at the indicated days. The logarithm of the mean number of cells is plotted against time. C. Effect of sericin on Mv1Lu cells proliferation was assessed by counting cells at the indicated days. The logarithm of the mean number of cells is plotted against time.
(TIF)

Figure S3 **MEK, PI3K and JNK inhibitors prevent fibroin and sericin cells migration stimulation.** Wound healing scratch assay was made on 24 hours serum-starved, confluent Mv1Lu cells that were treated with either 0.4% fibroin or 0.05% sericin for 24 hours, in the absence or presence of following inhibitors: SB431542, PD98059, LY294002, Y27632 and SP600125. A. Micrograph of the extent of closure obtained after 24 hours in the control sample or samples treated with fibroin supplemented or not with the indicated inhibitors. B. Micrograph of the extent of closure obtained after 24 hours in the control sample or samples treated with sericin supplemented or not with the indicated inhibitors. In all cases, the wounded area was examined by phase-contrast microscopy. C. Migration quantification was done in both A and B by measuring the gap area at 0 h (not shown) and after treatment. Cell migration was calculated and represented as the difference between the scratch area before treatment and the scratch area after treatment (see Materials and Methods). The experiment was repeated at least three times. A representative result is shown.
(TIF)

Figure S4 **Quantification of the c-Jun expression at the leading edge of the MDA-MB-231 control cells (A) or cells supplemented with either 0.4% fibroin (B) or 0.05% sericin (C).** The leading edge was divided in five sectors and the total fluorescence intensity was calculated in each sector. The graphs represent the average amount of fluorescence per nuclei in each sector (see Materials and Methods). The experiment was repeated at least three times. A representative result is shown.
(TIF)
