# Supplementary figures and images for: Fibroin and Sericin from Bombyx mori Silk Stimulate Cell Migration through Upregulation and Phosphorylation of c-Jun
Source: PLoS One. 2012 Jul 31;7(7):e42271. doi: 10.1371/journal.pone.0042271 (PMC3409175; doi:10.1371/journal.pone.0042271)

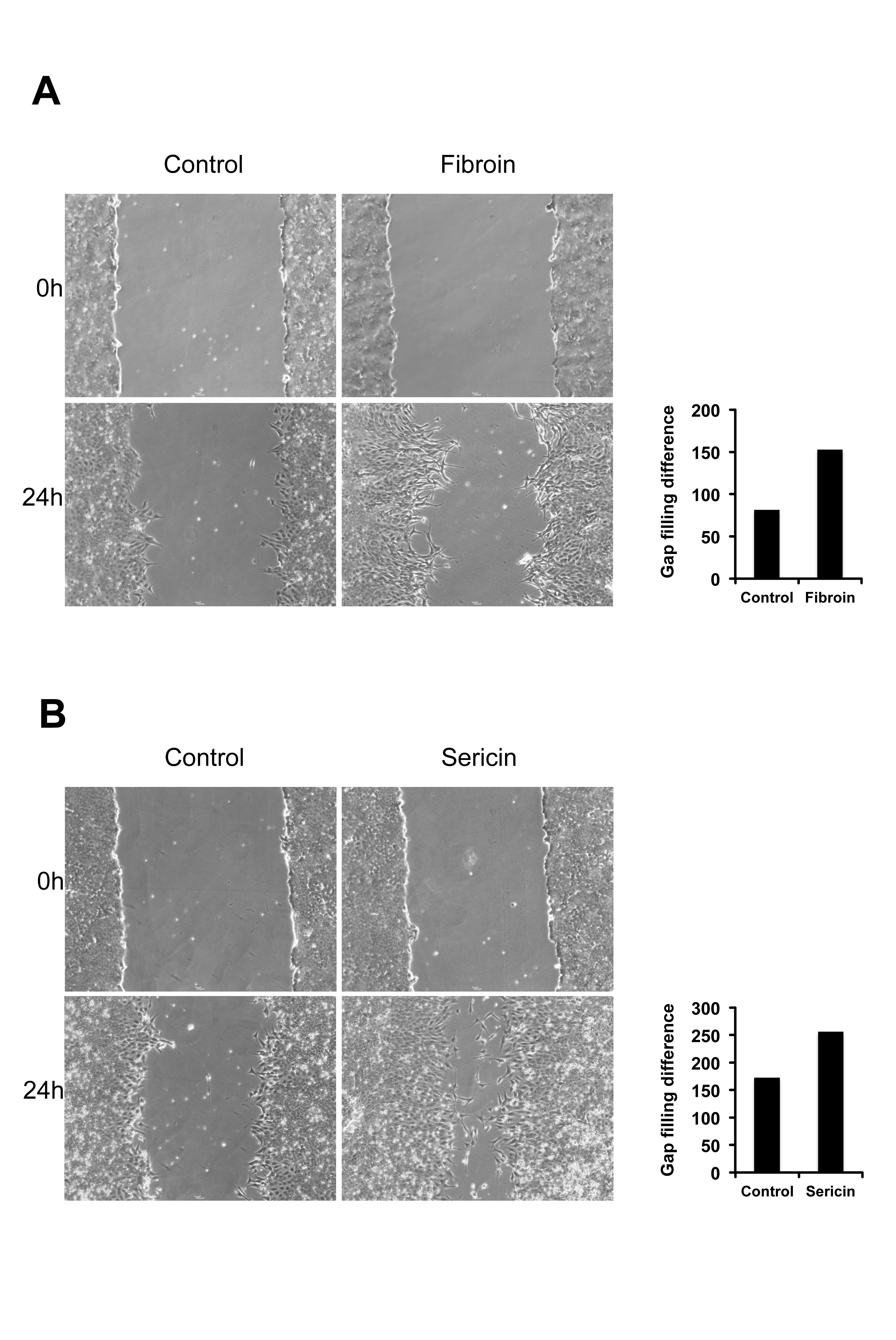

Supplement: Figure S1 — Fibroin and sericin induce motility in Mv1Lu cells. Wound healing scratch assay was made on 24 hours serum-starved, confluent Mv1Lu cells by drawing a line across the bottom of the dish, following by treatment with either 0.4% fibroin (A) or 0.05% sericin (B). The figure shows micrographs of the extent of closure obtained under control conditions compared to those with either fibroin or sericin after 24 hours treatment respectively. Phase-contrast microscopy pictures were taken of the wounded area. Migration quantification was done by measuring the gap area at 0 h and after treatment. Cell migration was calculated and represented as the difference between the scratch area before treatment and the scratch area after treatment (see Materials and Methods). The experiment was repeated at least three times. A representative result is shown. (TIF) [file pone.0042271.s001.tif]

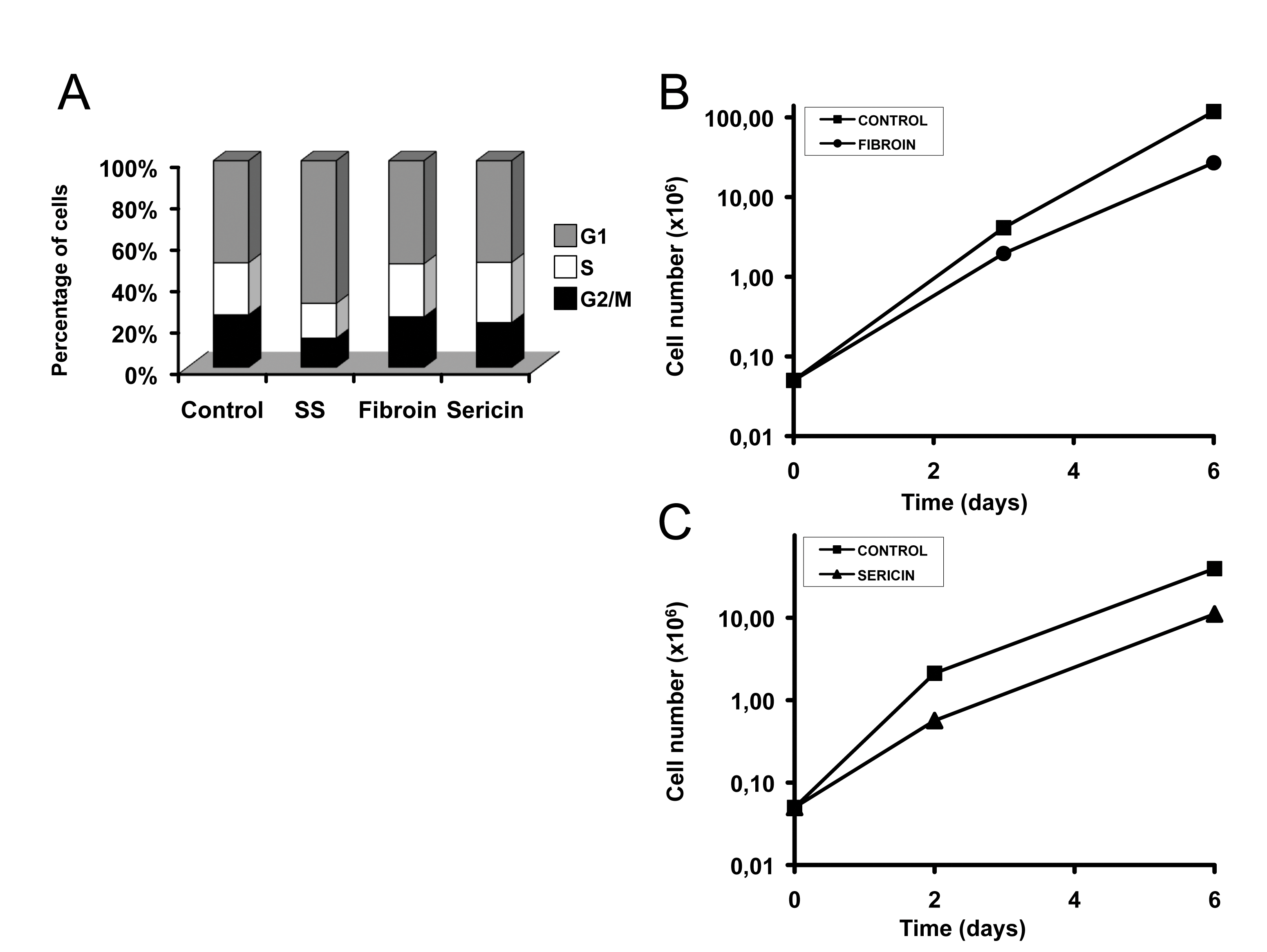

Supplement: Figure S2 — Fibroin and sericin do not affect cell cycle distribution or proliferation of Mv1Lu cells. A. Mv1Lu cells were treated with either 0.05% sericin or 0.4% fibroin, or serum starved (indicated as SS) for 24 hours. Cell cycle distribution was measured using fluorescence activated cell sorting (FACS). Data are presented as percentage of cells at the different phases of cell cycle. B. Effect of fibroin on Mv1Lu cells proliferation was assessed by counting cells at the indicated days. The logarithm of the mean number of cells is plotted against time. C. Effect of sericin on Mv1Lu cells proliferation was assessed by counting cells at the indicated days. The logarithm of the mean number of cells is plotted against time. (TIF) [file pone.0042271.s002.tif]

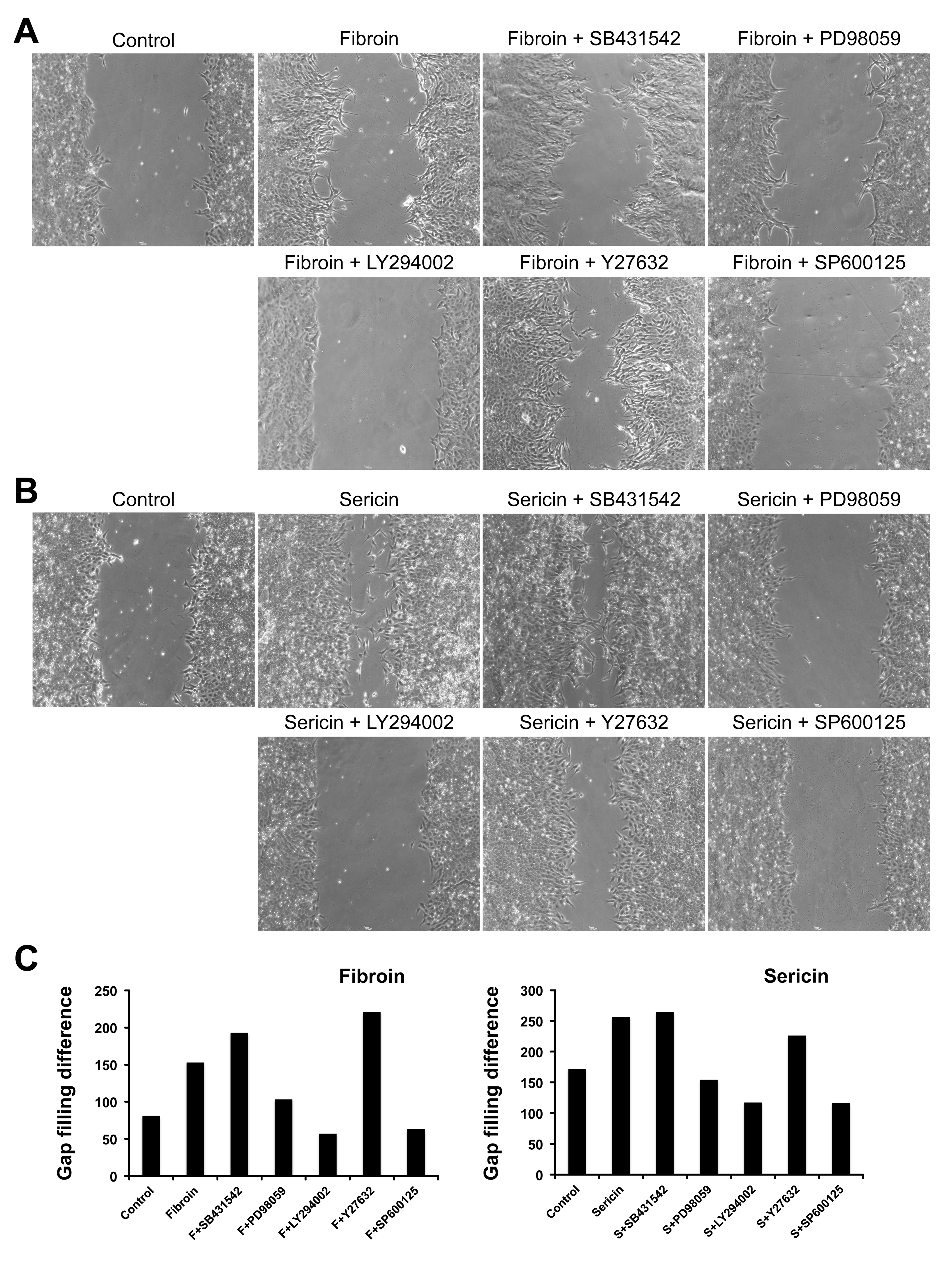

Supplement: Figure S3 — MEK, PI3K and JNK inhibitors prevent fibroin and sericin cells migration stimulation. Wound healing scratch assay was made on 24 hours serum-starved, confluent Mv1Lu cells that were treated with either 0.4% fibroin or 0.05% sericin for 24 hours, in the absence or presence of following inhibitors: SB431542, PD98059, LY294002, Y27632 and SP600125. A. Micrograph of the extent of closure obtained after 24 hours in the control sample or samples treated with fibroin supplemented or not with the indicated inhibitors. B. Micrograph of the extent of closure obtained after 24 hours in the control sample or samples treated with sericin supplemented or not with the indicated inhibitors. In all cases, the wounded area was examined by phase-contrast microscopy. C. Migration quantification was done in both A and B by measuring the gap area at 0 h (not shown) and after treatment. Cell migration was calculated and represented as the difference between the scratch area before treatment and the scratch area after treatment (see Materials and Methods). The experiment was repeated at least three times. A representative result is shown. (TIF) [file pone.0042271.s003.tif]

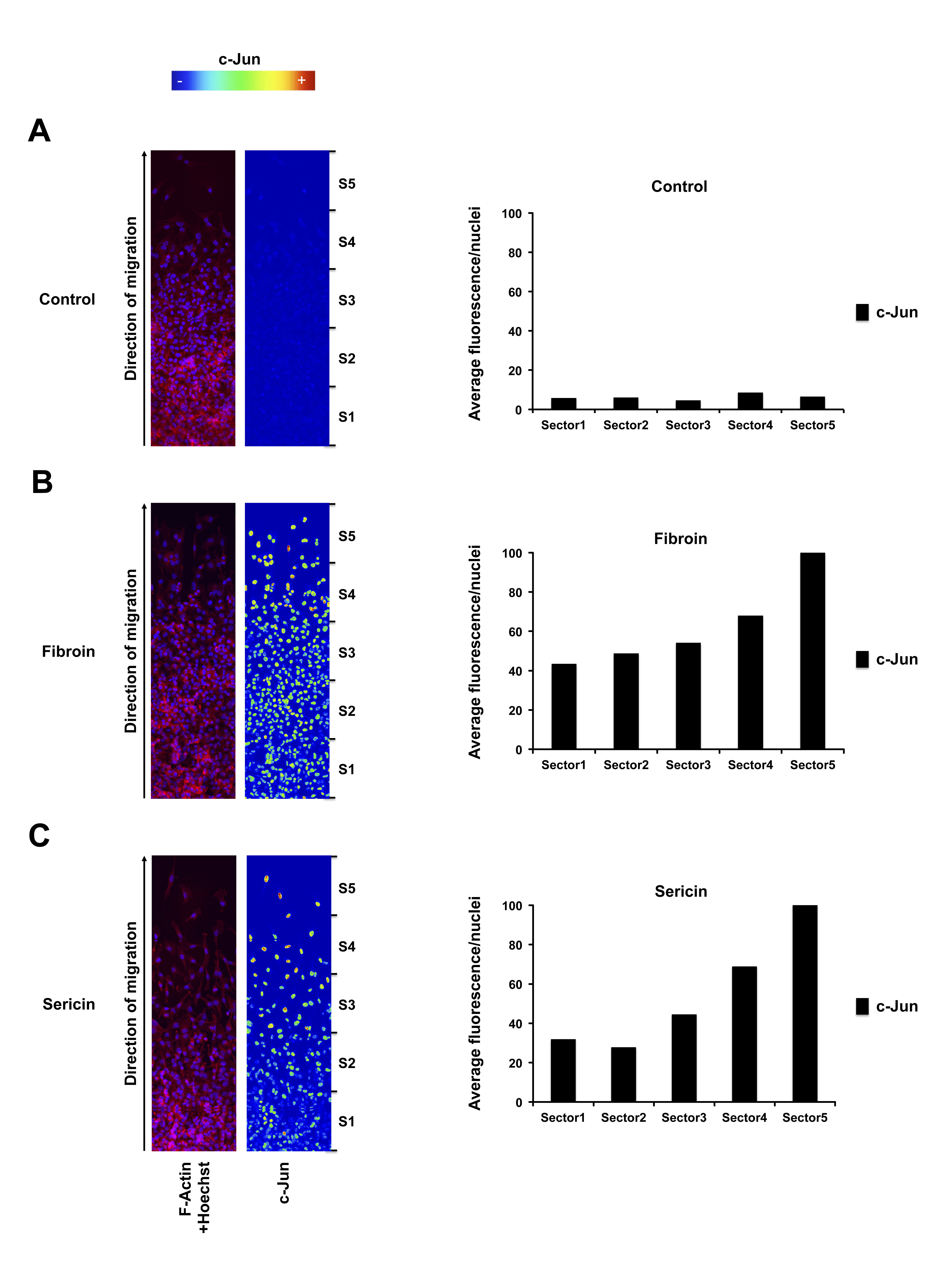

Supplement: Figure S4 — Quantification of the c-Jun expression at the leading edge of the MDA-MB-231 control cells (A) or cells supplemented with either 0.4% fibroin (B) or 0.05% sericin (C). The leading edge was divided in five sectors and the total fluorescence intensity was calculated in each sector. The graphs represent the average amount of fluorescence per nuclei in each sector (see Materials and Methods). The experiment was repeated at least three times. A representative result is shown. (TIF) [file pone.0042271.s004.tif]
